# Identification of barriers to the prevention and treatment of heat-related illness in Latino farmworkers using activity-oriented, participatory rural appraisal focus group methods

**DOI:** 10.1186/1471-2458-13-1004

**Published:** 2013-10-24

**Authors:** Michelle Lam, Jennifer Krenz, Pablo Palmández, Maria Negrete, Martha Perla, Helen Murphy-Robinson, June T Spector

**Affiliations:** 1 School of Medicine, University of Washington, Seattle, WA, USA; 2 Department of Environmental and Occupational Health Sciences, University of Washington, 4225 Roosevelt Way NE, Suite 100, Seattle, WA 98105, USA; 3 Department of Medicine, University of Washington, Seattle, WA, USA

**Keywords:** Heat-related illness, Heat exhaustion, Heat stroke, Agricultural workers, Farmworkers, Cultural beliefs

## Abstract

**Background:**

Heat-related illness (HRI) is an important cause of non-fatal illness and death in farmworkers. We sought to identify potential barriers to HRI prevention and treatment in Latino farmworkers.

**Methods:**

We conducted three semi-structured focus group discussions with 35 Latino farmworkers in the Central Washington, USA area using participatory rural appraisal techniques. Interviews were audio taped and transcribed in Spanish. Three researchers reviewed and coded transcripts and field notes, and investigator triangulation was used to identify relevant themes and quotes.

**Results:**

Although the majority of participants in our study reported never receiving formal HRI training, most participants were aware that extreme heat can cause illness and were able to accurately describe HRI symptoms, risk factors, and certain prevention strategies. Four main observations regarding farmworkers’ HRI-relevant beliefs and attitudes were identified: 1) farmworkers subscribe to varying degrees to the belief that cooling treatments should be avoided after heat exposure, with some believing that such treatments should be avoided after heat exposure, and others encouraging the use of such treatments; 2) the desire to lose weight may be reflected in behaviors that promote increased sweating; 3) highly caffeinated energy drinks are preferred to increase work efficiency and maintain alertness; and 4) the location of drinking water at work (e.g. next to restrooms) and whether water is clean, but not necessarily chemically-treated, are important considerations in deciding whether to drink the water provided at worksites.

**Conclusions:**

We identified potential barriers to HRI prevention and treatment related to hydration, certain HRI treatments, clothing use, and the desire to lose weight among Latino farmworkers. Strategies to address potential barriers to HRI prevention and treatment in this population may include engineering, administrative, and health education and health promotion strategies at individual, workplace, community, and societal levels. Although farmworkers in our study were able to describe HRI risk factors, reported practices were not necessarily consistent with reported knowledge. Further study of potential knowledge-behavior gaps may uncover opportunities for additional HRI prevention strategies. Farmworkers and employers should be included in the development and evaluation of interventions to prevent HRI.

## Background

Heat-related illness (HRI) is an important cause of preventable death globally [1,2]. HRI comprises a spectrum of disorders ranging from heat rash to heat stroke, which can be fatal. Unlike classical HRI, which occurs more commonly in the elderly, very young, and those with chronic medical conditions, exertional HRI can occur in young, otherwise healthy individuals with high metabolic output rates from increased workloads, particularly when working in hot and humid environmental conditions. The physiological response to dissipate heat and maintain a normal core body temperature (heat strain), which is overwhelmed in exertional HRI, can also occur in relatively cool environments, depending on the amount of metabolic heat produced and the degree of acclimatization of the worker [3,4]. Although classical HRI and exertional HRI in athletes and military personnel and have been studied extensively, less is known about exertional HRI in certain vulnerable working populations, including agricultural workers [5-9].

Between 2003 and 2008, the United States Agriculture, Forestry, and Fishing (US AFF) sector had the highest mean heat fatality rate, compared to all industries (approximately 0.3 deaths/100,000 full-time workers, compared to 0.02 for all industries), with the majority of fatalities occurring in the crop production and support subsectors [4,10]. Studies using US workers’ compensation claims data have identified a high burden of non-fatal HRI in the AFF sector despite probable substantial under-reporting [11]. The US AFF sector employs over two million workers, and about half of these workers are employed in the crop production subsector [12]. Hired farmworkers in the US are largely seasonal, foreign-born, Spanish-speaking workers [13].

Climate change threatens to increase the risk of HRI in farmworkers over time. Extreme heat is associated with heat-related deaths, and the frequency and intensity of heat waves is projected to increase locally and globally [14,15]. These findings indicate that the identification of risk factors for HRI in farmworkers, with the overall aim of HRI prevention, is timely and of public health significance.

Hydration and HRI-related cultural beliefs and practices in Latino farmworkers may affect the prevention and treatment of HRI. Previous studies in Latino communities have described distrust in the municipal water supply and of water provided in opaque containers, where the contents and cleanliness of water is difficult to determine [16-18]. In a qualitative study of farmworkers in Washington State, farmworkers expressed a belief that a cold shower should be avoided immediately after heat exposure, as cold water on a hot body could cause pain in the bones and joints [17]. In addition, *machismo* attitudes in certain Latino men may influence how HRI symptoms are addressed and communicated to peers and employers [8]. Although reports indicate that Latino farmworkers perceive HRI as an important health issue [7] and HRI knowledge and general beliefs have been studied [6,19,20], little has been done to systematically characterize cultural beliefs that may serve as barriers to HRI prevention in this population.

Certain US states, including California and Washington (WA), have adopted workplace safety standards intended to address outdoor heat exposure and prevent HRI [21,22]. The WA Agriculture Heat Rule, which applies to outdoor workers from May 1 through September 30 exposed to outdoor heat at or above specified temperature action levels, includes requirements for employers to address heat safety in the written accident prevention program [Washington Administrative Code (WAC) 296-307-09730-1-1]; encourage employees to frequently consume acceptable beverages (WAC 296-307-09730-1-2); ensure sufficient drinking water is accessible to employees and that employees have the opportunity to drink at least one quart of drinking water per hour (WAC 296-307-09740-1-1, 2); respond to employees with signs and symptoms of HRI (WAC 296-307-09750); and provide worker and supervisor HRI training (WAC 296-307-09760-1, WAC 296-307-09760-2) [21,22]. However, these standards impose generic rules that may not be equally protective in all agricultural settings and populations. For example, the exact amount of drinking water required for a particular employee to stay optimally hydrated may depend on several factors, including environmental conditions, metabolic heat production, and certain personal factors.

This formative study was performed as an initial stage in a larger US Centers for Disease Control and Prevention/National Institutes for Occupational Safety and Health (CDC/NIOSH)–funded study of risk factors for HRI in agricultural workers, conducted by researchers at the Pacific Northwest Agricultural Safety and Health (PNASH) Center at the University of Washington (UW). The objective of this study was to identify potential barriers to HRI prevention and treatment, including culturally-grounded beliefs, in order to inform the development of a validated survey of HRI risk factors which will be conducted among Latino farmworkers, along with environmental and physiological measurements, in later stages of the study.

This paper describes how participatory rural appraisal (PRA) focus group discussion methods were used to identify potential barriers to HRI prevention and treatment among Latino farmworkers in the Central WA, USA area, reports the results of the study, and discusses possible implications of these findings on the prevention of HRI.

## Methods

### Study sites and population

The study was conducted in the Central WA area, which is characterized by warm, dry summers and a productive agricultural sector. Climate and irrigation have contributed to the successful production of apples, pears, peaches, hops, cherries, grapes, blueberries, and other crops in WA. Between 1980 and 2006, the mean daily high Humidex (a measure of the combined effect of heat and humidity on human physiology) between May and September was approximately 25-28°C (77-83°F) [14]. The 99^th^ percentile of daily high Humidex annually was approximately 36-38°C (96-101°F), and the mean annual number of heat events (above the 99^th^ percentile) was 1.6. In an analysis of outdoor WA workers’ compensation agriculture and forestry HRI claims between 1995 and 2010, the mean daily difference in maximum and minimum temperatures by date and location of injury was 41.6°F [Spector et al., unpublished observations].

A purposive sample of farmworkers from the Central WA area was recruited to participate in the study during the spring of 2012. Research staff first developed collaborative agreements with local agricultural businesses using established PNASH contacts. Participants were then recruited from these businesses’ worksites by Spanish bilingual and bicultural research staff. Participants were eligible if they were adult (age 18 or older) workers who conducted outdoor summer farm work in the Central WA area. Recruitment was not restricted by gender or minority status.

Spanish bilingual and bicultural research staff conducted three focus group discussions in Spanish using semi-structured interviewing techniques and PRA methods. PRA methods include participant-generated visual diagrams and maps, direct observations and journaling of local conditions, analytic games, storytelling, and seasonal calendars [23]. These methods have been successfully used in farming field research to generate data to tailor health services and education programs to agricultural workers [24]. PRA methods were used to better engage our study population and enhance data collection in a manner similar to the addition of activity-oriented questions to focus groups discussions that has been previously described [25]. However, since we used methods adapted from PRA, we describe our focus groups as PRA focus group discussions.

During PRA focus group discussions, participants in this study were asked about knowledge and practices related to HRI symptoms and associated risk factors, treatments, prevention, and hydration. We deduced information about beliefs from participant discussion of practices when beliefs were not specifically verbalized. A PRA facilitator’s guide, developed prior to the focus group discussions, was used to standardize discussion across the three sessions (Additional file 1). For consistency, all sessions were led by the same research staff member (P.P.). The size of the focus groups ranged from 11 to 12 people each, and the length of the focus groups discussions ranged from 182 minutes to 233 minutes, with a mean length of 209 minutes. PRA focus groups discussions were held at participating worksites or the PNASH field office in Yakima, WA.

As an incentive for worksites to participate in the study, we offered HRI education required by the WA Agriculture Heat Rule (WAC 296-307-097) [21]. In addition, all focus group participants were offered a $40 gift card to account for missed work time while participating in the study. The UW Institutional Review Board approved the study protocol, and each participant provided written informed consent prior to participation.

### Data collection

Demographic information was collected at the beginning of each focus group discussion using questions that were administered in Spanish either with an electronic audience response system (TurningPoint Anywhere version 3.0.2.1171, Turning Technologies, LLC, Youngstown, Ohio), which allows participants to enter individual responses, or self-administered on paper. All demographic questions were read aloud by research staff, and staff members were available to help participants answer these questions.

**Figure 1:**
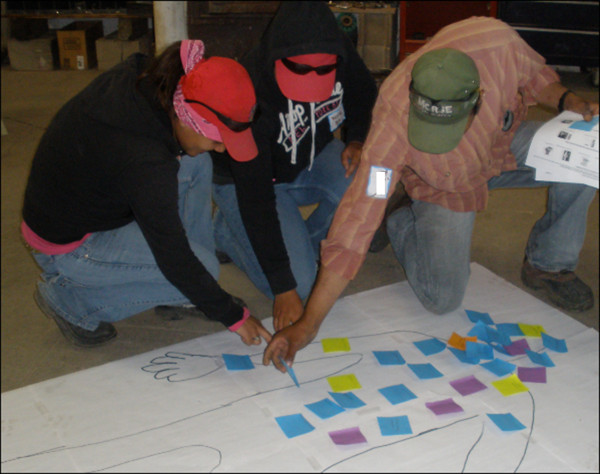
Body mapping to assess beliefs about HRI symptoms.

**Figure 2:**
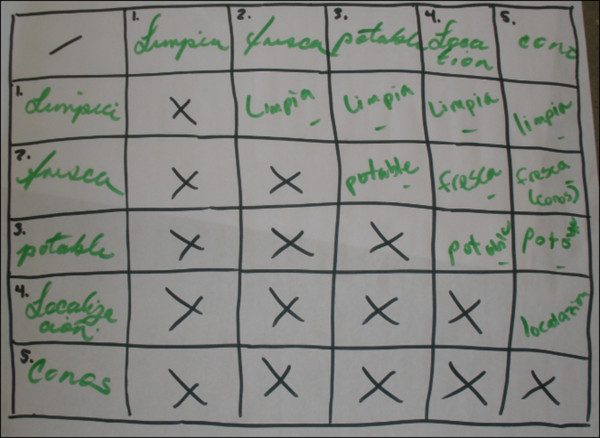
Priority grid approach to assess preferred drinking water characteristics.

Knowledge, beliefs, and practices regarding HRI causes, treatments, and prevention were assessed by having participants write and draw, organize notes, drawings, and ideas, and explain to the group what they believed to be HRI causes, treatments, and prevention strategies. Signs and symptoms of HRI were assessed using PRA body mapping techniques [26]. Participants were asked to draw an outline of a body and indicate on the body map what they believed to be HRI symptoms and the corresponding body part affected (Figure 1). Preferred characteristics of drinking water were assessed using a pairwise-ranking approach [27], in which different combinations of participant-generated characteristics of water were compared and prioritized by the group (Figure 2).

All PRA focus group discussions were audiotaped. Participant responses were also recorded using written notes that participants placed on PRA diagrams and charts.

### Data analysis

PRA focus group discussions were transcribed in Spanish. Utilizing investigator triangulation, three researchers (M.L., M.P., M.N.) reviewed the transcripts and field materials in Spanish to identify patterns that may be potential barriers to HRI prevention and treatment. Codes to describe these themes and patterns were discussed, agreed upon by consensus by the three researchers, and recorded. Atlas.ti Version 7.0 software (Scientific Software Development, Berlin) was used to code and categorize observations. Demographic characteristics were summarized using descriptive statistics and presented as overall totals for the whole study group and stratified by focus group.

## Results

Thirty-five workers participated in the study. Demographic characteristics of participants are shown in Table 1. Three focus groups discussions were completed in blueberry (n=11), peach (n=12), and apple and cherry (n=12) workers. The majority (60%) of participants were male; all participants in the first focus group discussion were female, all participants in the third focus group discussion were male, and both male and female workers participated in the second focus group discussion. The majority of participants were born in Mexico (97%), were between 26 and 45 years of age, and had been living in the US for more than 10 years at the time of the study. A minority of participants had more than a 9^th^ grade education (19%). Only two participants reported having previously received formal HRI training. All but one participant reported believing that extreme heat can cause illness, and participants accurately reported most HRI symptoms, including symptoms consistent with heat rash, heat exhaustion, heat cramps, and heat syncope. Four main observations reflecting potential barriers to HRI prevention and treatment related to hydration, cooling treatments, clothing use, and the desire to lose weight were identified during the PRA focus group discussions (Table 2). Further details of results regarding HRI risk factors, hydration, and HRI treatment and prevention are presented in the sections below.

**Table 1:** Demographic characteristics by focus group

**Demographic characteristic**	**Focus group 1 (n=11)**	**Focus group 2 (n=12)**	**Focus group 3 (n=12)**	**Total N (%)**
Gender				
Male	0	9	12	21 (60%)
Female	11	3	0	14 (40%)
Age (years)*				
<25	2	2	1	5 (16%)
26-35	6	3	3	12 (34%)
36-45	1	4	5	10 (29%)
> 45	1	1	3	5 (16%)
Country of birth*				
Mexico	9	10	12	31 (97%)
United States	1	0	0	1 (3%)
Years lived in United States*				
<1	0	0	0	0 (0%)
2-4	0	1	0	1 (3%)
5-7	2	1	0	3 (9%)
8-10	2	4	4	10 (29%)
>10	6	4	8	18 (56%)
Highest grade of school*				
Did not complete primary school	2	2	0	4 (13%)
Primary school (grade 1–6)	1	4	5	10 (29%)
Some middle school (grade 7–9)	4	4	4	12 (38%)
High school	3	0	2	5 (16%)
Greater than high school	0	0	1	1 (3%)
Crop				
Blueberries	11	0	0	11 (31%)
Peach	0	12	0	12 (34%)
Apples/cherries	0	0	12	12 (34%)

* Three values missing (one from Focus Group 1; two from Focus Group 2). Percentages for these categories are out of 32 instead of 35.

**Table 2:** Overview of main observations noted during participatory rural appraisal focus group discussions with Latino farmworkers

	
•	Farmworkers subscribe to varying degrees to the belief that cooling treatments should be avoided after heat exposure, with some believing that such treatments should be avoided after heat exposure, and others encouraging the use of such treatments.
•	The location of water at work (e.g. next to restrooms) and whether water appears clean, but not necessarily chemically-treated, are important considerations in deciding whether to drink the water provided at worksites.
•	Highly caffeinated energy drinks, such as Monster® and Red Bull™, are strongly preferred to increase work efficiency and maintain alertness.
•	The desire to lose weight may be reflected in behaviors that promote increased sweating.

### HRI risk factors

#### Hydration

Participants described not drinking enough water as a cause of HRI. Both male and female participants reported that they “do not always have water with them while working,” which could lead to “dehydration…dizziness…and headaches.” One female participant remarked, “We can get sick if we don’t bring water to work.” Both male and female participants also identified “drinks with a lot of caffeine” and “drinking alcohol” as causes of HRI. Participants reported knowing that they were dehydrated if they “were not sweating,” “lacked energy to work,” “felt nauseated or dizzy,” or “felt that their skin was looser.”

#### Clothing

Participants reported that wearing “dark or tight clothing” could cause HRI. One female participant reported that “dark clothing….burns more.” However, participants also reported wearing darker clothes in order to sweat more and lose weight: “If you want to sweat….if you want to….burn fat….dark colors give out more heat.” Female participants reported wearing “Lycra® leggings” and “short-sleeved shirts” underneath their clothes to stay warm during the cooler early hours of the work day. However, they noted that they often did not take all extra layers off as the day became progressively warmer. Several participants noted that they wore sweatshirts layered on top of other short-sleeved shirts to “keep them cool [with their] own sweat.”

Male participants reported wearing back support belts and female participants reported wearing girdles under their clothing for back support during the harvest. Participants reported that men use support belts to provide back support and prevent hernias when lifting. Several participants noted that women thought they lost weight when wearing the girdles, which was an incentive to wear them in hot weather: “Sometimes ….even though it’s hot…the more you sweat the more you like it because you lose weight.”

Participants of both genders described that “not wearing enough clothing” or “not using a hat” could lead to sunburns; however, others noted that wearing too much clothing to protect from sunburns could cause overheating. Some participants reported using sunscreen to prevent sunburns. However, some female participants described that “sunscreen made them feel warmer” and thus they “would never wear it.” Participants noted that “long-sleeved blouses,” “light-colored sweatshirts,” and “hats” were worn to cover the body and “protect from sunburns.”

A female participant described that wearing protective clothing to reduce exposures to dust and chemicals contributes to HRI: “Some colleagues are all covered up…covered up to the nose…some people get sick because there is too much dust, chemicals, and pollen and you have to be covered from the mouth and nose…It can produce allergies, the eyes, the sneezing. It causes bronchitis similar to asthma and I feel like I can’t breathe. Being covered up can cause heat illness….breathing in the hot air.” Participants also reported wearing denim jeans because they are “thicker…and won’t get caught on tree branches.”

#### Environmental conditions & work characteristics

Participants of both genders noted that working “long hours in the hot sun and high temperatures” contributed to HRI. Participants reported that “not taking breaks in the shade can cause heat illness” and noted a lack of shade in the crop areas in which they work. Several women working in blueberry fields described how their work in areas without shade made them feel much warmer. Both male and female participants also identified other factors, such as excess work and working quickly, as causes of HRI.

#### Personal risk factors

Participants reported that chronic conditions, including high blood pressure and being overweight can contribute to HRI. Participants also noted that fatigue, lack of sleep, lack of physical fitness, and a poor diet could play a role in the development of HRI. Many participants reported that medications play a role in HRI. However, when asked about medications that increase the risk of HRI, participants recalled being told that exposure to sunlight was not advised when taking certain medications, such as medications for bladder infections. Medications that lead to dehydration, increase metabolism, inhibit sweating or blood vessel dilation, or reduce heart rate and/or contractility were not specifically mentioned.

### Hydration

#### Beverage types

Participants reported that Gatorade® is a popular beverage to help “replace the electrolytes lost while sweating.” Participants also reported drinking Crystal Light™ “to help flavor….water.” Female participants reported drinking certain local drinks, such as ‘agua de Jamaica’ (hibiscus water) or ‘agua de arroz’ (rice water) “to help refresh the body and stomach.” Several men also discussed “bringing hidden beer” to the worksite to help overcome thirst.

To help “stay alert…work faster…and fatigue more slowly,” participants reported drinking soda, energy drinks (such as Monster® or Red Bull™), and coffee. However, participants of both genders acknowledged that water is the healthiest beverage to consume at work. Workers reported that water is the only beverage provided by employers; all other beverages were brought from home by the workers, or bought from a stand or store.

#### Frequency of hydration

Most participants discussed drinking beverages at different times of day – either “during breaks” or “at the end of working.” Reasons for not drinking water more frequently included not wanting to interrupt work in order to avoid upsetting their supervisor and to make more money (e.g. for piece rate workers). One participant noted: “sometimes we don’t drink water so we won’t have to use the bathroom, because it’s too far away.” Male and female participants noted they drank water when they were thirsty, but whether they were thirsty depended on how hot it was outside. Some participants talked about drinking beverages every hour.

#### Water characteristics

For many participants, water cleanliness was the most important water characteristic, compared to freshness, potability, location, or source. Participants of both genders seemed to make a clear distinction between clean water and potable water. Participants described clean water as “water that appeared clear and not cloudy,” but was not necessarily potable. Participants described potable water as “water that was chemically treated.” Participants of both genders stated that they preferred “clear water” to “chemically treated water,” indicating that they did not like the taste of chemically treated water. “Freshness” referred to water that was frequently changed in water jugs at worksites. Several female participants described water that is “clean….[and]….changed daily” as the most desirable water to drink at work.

#### Location of water

Female participants noted that the location of water next to the bathroom at the worksite was problematic. Participants described that men would often urinate or otherwise contaminate the drinking water located near the bathroom: “[Water at work is] next to the bathroom….I don’t think it’s healthy….that you would be drinking this….the water container [should] not be exposed to a person who might want to do some bad….get….some trash, dirt, or wash their hands in it….or urinate….sometimes that is what the men do.”

### HRI treatment & prevention

#### Fluids & electrolytes

Several male participants identified fluids, such as water, Gatorade®, lemonade with salt, and intravenous fluids as treatments for HRI. One male participant advised giving “electrolyte pills that dissolve in water” as another treatment. Both male and female participants noted that beer or tea are not appropriate treatments for someone suffering from HRI.

#### Cooling treatments

Several female participants described “placing ice packs on the forehead and neck, or top of head…to refresh…[workers suffering from HRI].” In contrast, several other female participants reported that they learned from their families that “you should not put cold water on the body because it can cause headaches or you might also faint.” A male participant stated that he “would not give a bottle of cold water to a person who is suffering from heat.” Female participants noted that “when you are….working in the sun, moving your hands a lot….you can get arthritis [if]….you get home and you wet them,” and “when the body is hot, they say it’s bad to drink very cold water because you can develop blisters inside the mouth.”

Participants stated they would “loosen tight clothing” and “remove excess layers” as HRI treatments. Fanning the person “with air” was also suggested to help a person suffering from HRI to cool down.

#### Other treatments

Participants recommended “stopping work” and “sitting in the shade” to treat someone who was suffering from HRI. Several participants of both genders noted they would “check the pulse,” “call 911,” and “inform a supervisor, employer, or other co-worker” to get help for a person who might be suffering from HRI. Several participants discussed the importance of “not surrounding or agitating a person” that may be suffering from HRI. If someone was unconscious due to heat stroke or heat syncope, participants described how they would use “smelling onions” or “alcohol” to help reawaken the afflicted person. Strong coffee without sugar was suggested by one participant as a treatment for HRI.

#### Prevention

In addition to hydration, participants of both genders recommended wearing “clothing that is light and not too thick to be comfortable while at work and not give off too much heat.” They also recommended that clothing “should not be tucked in…to allow for more ventilation.” Shade was mentioned several times as an important method of prevention of HRI.

## Discussion

In this qualitative study of Latino farmworkers in the Central WA, USA area using PRA focus group discussion methods, potential barriers to HRI prevention and treatment related to hydration, certain HRI treatments, clothing use, and the desire to lose weight were identified. Proposed strategies to address these potential barriers, and the type and scope of these strategies, are shown in Table 3.

**Table 3:** Strategies to address barriers to heat-related illness prevention and treatment identified during participatory focus groups

**Potential barriers**	Proposed strategies to address barriers*	**Strategy type/scope**
**Behaviors reflecting cultural beliefs**	Add the following information to train-the-trainer HRI educational materials:	Education/workplace & individual
E.g. Avoidance of certain HRI treatments	1) a review of the potential role of cultural beliefs, such as beliefs related to cooling treatments after heat exposure, in the prevention and treatment of HRI;	
	2) recommendations for trainers to identify and, if present, acknowledge the role of cultural beliefs in a non-judgmental and respectful manner;	
	3) an explanation of rapid cooling treatments for workers with heat stroke;	
	4) recommendations for trainers to involve workers in developing effective and culturally acceptable strategies for treating workers with heat stroke	
**Competing health priorities**		
E.g. Weight loss (via sweating); Back injury prevention	Direct workers to community-based obesity prevention and fitness programs, if available, or integrate elements of such programs into workplace health promotion activities.	Health promotion/ community & workplace
**Competing workplace hazards & controls**		
E.g. Non-breathable chemical resistant suits for pesticide handlers;	Develop and use more breathable chemical-resistant suits;	Personal protective equipment/workplace
Prevention of ultraviolet light (UV) exposure	Enhance UV protection of light-colored, breathable clothing by frequent laundering with ultraviolet absorbent agents, or use clothing with pre-integrated UV protection;	
	Encourage sunscreen use during worker HRI training	Education/individual
**Hydration & workplace factors**		
E.g. Inadequate hydration due to lost wages from taking breaks among piece rate workers, negative reactions from supervisors regarding water breaks, lack of nearby bathroom facilities;	Implement a standardized system of water break reminders at reasonable intervals on days with high heat/humidity;	Administrative/ workplace
	Implement salaried or hourly payment schemes instead of piece rate;	
	Locate bathroom facilities close to workers;	
Water does not appear clear and is not changed regularly;	Adhere to basic field sanitation requirements (drinking water is provided in a closable container, is clearly labelled as such in a language that workers can understand, is readily accessible to workers, has a tap, is suitably cool, and containers are refilled regularly);	
Water is located in opaque containers, near bathrooms (perceived as contaminated);	Locate water away from restrooms (but near workers) in non-opaque containers;	
	Arrange for an employee to deliver water to workers at regular intervals using an all-terrain or other vehicle;	
Energy drinks preferred to increase alertness and productivity	Provide preferred, recommended beverages; Include information on sleep hygiene and fatigue mitigation in health promotion activities	Health promotion/ community & workplace
**Other workplace factors**		
E.g. Lack of shade	Add workplace shade requirements to regulations, if not already included	Engineering (shade)/ societal

HRI, heat-related illness; UV, ultraviolet.

*Employers and workers should be involved in the discussion and development of acceptable interventions to prevent HRI.

Although most participants reported an awareness of heat health effects and were able to describe HRI risk factors, reported practices were not necessarily consistent with reported knowledge. For example, workers identified dark, tight clothing as a cause of HRI and removal of layers as an HRI treatment, but female participants also reported wearing darker clothing to sweat to lose weight and wearing sweatshirts layered on top of short-sleeved shirts to induce sweating. Alcohol was recognized as a cause of HRI and water as the healthiest beverage to consume at work, but some participants noted bringing beer to work to help quench thirst, and participants reported drinking highly caffeinated energy drinks to increase alertness and productivity. These discrepancies may have resulted from certain attitudes and beliefs, including cultural beliefs, competing health priorities and workplace hazards and controls, and a lack of perceived ability to exert personal control over certain HRI risk factors [28].

### HRI knowledge

Although the majority of participants in our study reported never receiving formal HRI training, most participants were aware that extreme heat can cause illness. These findings are consistent with other studies, including a study of migrant and seasonal farmworkers in Michigan USA, where heat exhaustion was identified as a commonly-perceived health problem by many participants [7]. Among 474 hired farm workers in the Mexican Immigration to California: Safety and Acculturation (MICASA) Study, the majority of whom had received HRI training (91.6%), over half of participants were at least a little bit concerned about the risk of HRI at work [20].

Most participants in our study accurately described HRI symptoms and causes. Participants mentioned feeling “dizzy” if they did not drink enough water. Decrements in vigilance and endurance during heat exposure have also been described [29], which could increase the risk of falls from ladders and other equipment. Several epidemiologic studies have suggested that there is a relationship between occupational heat stress and injury [30,31].

Many participants correctly identified a lack of hydration with appropriate beverages, wearing dark-colored clothing, high exertion (excess work and working quickly), and certain personal factors as risk factors for exertional HRI. Although we did not quantify HRI risk factors in this qualitative study, our findings are consistent with those previously reported: HRI knowledge assessed in the MICASA study using a standardized survey instrument was moderate [20].

Participants in our study were able to correctly identify certain HRI prevention strategies and treatments, including hydration with appropriate beverages, removing clothing layers, resting in the shade, and reporting and getting help for affected workers. However, participants also mentioned drinking alcohol to quench thirst and strong coffee as an HRI treatment, which are not recommended. These behaviors and misconceptions could be addressed during worker and supervisor HRI training, which is required in certain states, including WA (WAC 296-307-097) [21].

### Cultural beliefs & cooling treatments

Some participants reported believing that headache, fainting, arthritis, and oral blisters may be caused by exposure to cold immediately after heat. Failure to recognize and address beliefs such as these could lead to less effective heat stroke treatment, which involves rapid cooling and reduction of core body temperature to prevent death [32].

Barriers to effective, culturally sensitive education regarding HRI treatment may include educators’ and trainers’ lack of awareness or negative reactions to workers’ cultural beliefs [33]. To begin to address these barriers, the following information could be added to train-the-trainer HRI educational materials: 1) a review of the potential role of cultural beliefs, such as beliefs related to cooling treatments after heat exposure, in the prevention and treatment of HRI; 2) recommendations for trainers to identify and, if present, acknowledge the role of cultural beliefs in a non-judgmental and respectful manner; 3) an explanation of rapid cooling treatments for workers with heat stroke; and 4) recommendations for trainers to involve workers in developing effective and culturally acceptable strategies for treating workers with heat stroke.

### Competing health priorities

Participants raised two main health priorities that may interfere with HRI prevention. One health priority was weight loss. Although participants had an awareness that tight, dark clothing could contribute to HRI, some participants, particularly women, noted that wearing dark clothing and girdles under their clothing led to sweating and weight loss. While sweating can lead to a decrease in water weight, it can also lead to dehydration and HRI. The relatively large diurnal temperature variation in WA may contribute to workers’ behaviors of wearing extra layers during earlier cooler parts of the work day in order to stay warm. However, workers reported that they often did not take all extra clothing layers off as the day became progressively warmer, potentially increasing the risk of HRI.

The second health priority was back pain prevention. Some male participants reported wearing back support belts to prevent back injury. However, there is little evidence for the effectiveness of back support belts in primary or secondary prevention of back pain [34], and the belts could contribute to trapping heat and preventing evaporative cooling. Directing workers to community-based obesity prevention and fitness programs [35], if available, or integration of elements of such programs into workplace health promotion activities, may be one way to address healthy weight loss, fitness, and back injury prevention without increasing the risk of HRI.

### Competing workplace hazards and controls

Participants reported several competing workplace hazards and controls that may serve as barriers to HRI prevention. First, participants noted that wearing personal protective equipment (PPE) to protect from dust, allergens, and chemicals could increase the risk for HRI. Participants also noted that they preferred wearing thicker denim clothes to avoid being injured by tree branches. Although the WA Agriculture Heat Rule does require worker education on the importance of removing heat-retaining PPE during all breaks [21], development of PPE that is protective but retains less heat may be beneficial. For example, development and use of more breathable chemical-resistant suits for pesticide handlers could help address both hazards. Lightweight material with a repellent finish was evaluated during field trials as PPE for use in hot climates and may be appropriate when using certain pesticides [36]. However, further testing is needed.

Finally, some participants noted that they preferred to wear clothing with greater skin coverage to prevent ultraviolet (UV) light exposure, resultant sunburn, and darkening of the skin, which is perceived as undesirable, particularly by women. However, additional clothing can also trap heat and prevent cooling. In addition to sunscreen, light-colored breathable clothing with integrated UV protection could be explored as a means to address this competing hazard. Although the US Occupational Safety and Health Administration already requires employers to pay for certain PPE, clothing with UV protection is not currently included in the scope of this regulation [37]. More practical approaches may include: 1) frequent laundering; and 2) addition of UV absorbent agents and detergents during laundering of white cotton garments, which have been shown to increase UV protection factors by 17-51% and 407%, respectively [38].

### Hydration & workplace factors

Participants’ reports of hydration frequency suggested that they may not be drinking enough water to stay adequately hydrated. Several participants reported drinking when they became thirsty. However, thirst cannot be relied upon as a guide for the need for water [32], as 1% of the total body weight in water is already typically lost when an individual senses thirst. Low self-reported water consumption has been observed in other studies in US farmworkers, and hypotheses regarding barriers to water consumption include lost wages from taking breaks among piece rate workers, negative reactions from supervisors regarding water breaks, and lack of nearby bathroom facilities [20]. Employer implementation of a standardized system of water break reminders at reasonable intervals, location of bathroom facilities closer to workers, and use of salaried or hourly instead of piece rate payment schemes may help address these barriers.

In our study, participants reported that a clear appearance of water and regular changing of water are most important when deciding whether or not to drink water provided at work. Female participants suspected that water located near bathrooms was contaminated. Previous studies in Latino farmworkers have indicated that water provided in opaque containers, where the contents and cleanliness of water is difficult to determine, is not trusted [17]. Although the WA Agriculture Heat Rule does require employers to ensure that all employees have the opportunity to drink at least one quart of water per hour [21], workers may be more likely to stay adequately hydrated if: 1) water is not located next to restrooms (but restrooms are readily available); 2) water is provided in closed non-opaque containers; and 3) basic field sanitation requirements are met (drinking water is clearly labelled as such in a language that workers can understand, is readily accessible to workers, has a tap, is suitably cool, and containers are refilled regularly). Another approach could be to encourage employers to have water delivered to employees at regular intervals by an employee who drives to work areas in an all-terrain or other vehicle supplied with water. This approach has been observed on an agricultural operation in Central WA, although it has not yet been assessed for effectiveness and acceptability among employers and employees.

Participants acknowledged that water is the healthiest beverage to consume at work. However, they also reported drinking energy drinks (such as Monster® or Red Bull™) to increase alertness and productivity. Although caffeine is not recommended for workers at risk for HRI due to its diuretic and stimulant effects [39], this view is somewhat controversial [40-42]. Providing water in a manner that is desirable to workers or providing preferred, recommended beverages, may encourage workers to stay hydrated with recommended beverages. Including information on sleep hygiene and fatigue mitigation in education and health promotion activities may also reduce workers’ perceived need for energy drinks.

### Other worksite factors

Several participants noted a lack of shade in the crop areas in which they work. Although the California USA Heat Rule addresses shade [22], the WA Agriculture Heat rule does not [21]. Addition of workplace shade requirements to regulations should be considered.

### Employer involvement

Employers and supervisors, in addition to workers, should be involved in the discussion and development of acceptable HRI prevention strategies for agricultural workplaces. Many HRI prevention strategies, including the strategies proposed in Table 3, require employer support and involvement. Farmworkers likely realize that workers have minimal control of certain HRI risk factors, such as workplace shade availability and proximity to bathroom and water facilities at work. Engaging with employers in the discussion and development of HRI interventions is likely to increase the chance of effectively addressing barriers to HRI prevention and treatment. Such participatory approaches that include employers and workers have been successfully used to develop practical solutions for pesticide safety in agricultural settings [43].

### Strengths & limitations

Strengths of this study include the use of PRA techniques, which allowed for active participant involvement and enabled participants to generate much of their own data. In addition, employers were not present during these sessions, giving participants a more open environment in which to participate. Leadership of sessions was conducted by the same research staff member using a facilitator’s guide, which allowed for standardization and consistency across sessions. Another strength of the study was the use of multiple team members to code and interpret transcripts and field materials in Spanish, which increases the likelihood of valid findings. Both the PRA session leader (P.P.) and one of the team members that participated in data analysis and interpretation (M.N.) are members of the Latino community in the Central Washington area, where our study took place, and have personal experience performing fieldwork. Their participation contributed to effective engagement of our study population and to validation of study observations. Finally, the study addressed not only participants’ knowledge and practices, but also relevant attitudes and beliefs.

Limitations of the study include the use of a purposive sample of participants, which may limit the generalizability of findings. Over half of our study group reported living in the US for over 10 years. Our participants may therefore be more acculturated than Latino farmworkers who have more recently moved to the US. In addition, the PRA focus group discussions included sections of HRI education, which could lead to bias in participant responses. However, educational exercises always occurred after participants shared their comments about HRI topics. Another limitation is that, for most reported beliefs, we did not delve into the underlying reasons for these beliefs. Our study was not designed to specifically compare male and female participant responses. We also did not measure the amount of time working in agriculture or specifically address acclimatization. The relatively large diurnal temperature variation in WA may contribute to suboptimal acclimatization, and previous studies have suggested that poor acclimatization in the setting of a heat event or a sudden increase in exertion may increase the risk of HRI in WA workers [11]. Finally, in this qualitative, hypothesis-generating study, we did not quantify participants’ responses or obtain objective data on practices and behaviors. We did not validate reported practices using field observations.

## Conclusions

We identified potential barriers to HRI prevention and treatment related to hydration, certain HRI treatments, clothing use, and the desire to lose weight among Latino farmworkers. Strategies to address potential barriers to HRI prevention and treatment in this population may include engineering, administrative, and health education and health promotion strategies at individual, workplace, community, and societal levels (Table 3) [4,44]. Evaluation of the effectiveness of these interventions is needed.

Although Latino farmworkers in our study were able to describe HRI risk factors, reported practices were not necessarily consistent with reported knowledge. Further study is needed to elucidate: 1) why knowledge of HRI symptoms and risk factors may not necessarily translate into such practices as adequate hydration with recommended beverages, wearing optimal clothing in hot conditions, and rapid cooling of individuals with HRI; and 2) how knowledge-behavior gaps may be influenced by certain cultural beliefs, competing health priorities and workplace hazards, and a lack of perceived ability to exert personal control over certain HRI risk factors. A better understanding of HRI-relevant knowledge-behavior gaps among farmworkers may lead to the development of additional strategies for HRI prevention.

## Abbreviations

(AFF): Agriculture, Forestry, and Fishing; (CDC): Centers for Disease Control and Prevention; (HRI): Heat-related illness; (MICASA): Mexican Immigration to California: Safety and Acculturation; (NIOSH): National Institutes for Occupational Safety and Health; (PRA): Participatory rural appraisal; (PNASH): Pacific Northwest Agricultural Safety and Health; (PPE): Personal protective equipment; (US): United States; (UV): ultraviolet; (UW): University of Washington; (WA): Washington State.

## Competing interests

The authors declare that they have no competing interests.

## Authors’ contributions

ML participated in PRA focus groups, led data analysis efforts, and participated in manuscript writing and revision. JK organized, contributed to the design of, and participated in PRA focus groups and participated in manuscript revision. PP led PRA focus groups and participated in manuscript revision. MN participated in PRA focus groups, data analysis, and manuscript revision. MP participated in PRA focus groups, data analysis, and manuscript revision. HMR participated in PRA focus group design and manuscript revision. JTS participated in study design, PRA focus groups, and manuscript writing and revision. All authors read and approved the final manuscript.

## Pre-publication history

The pre-publication history for this paper can be accessed here:

http://www.biomedcentral.com/1471-2458/13/1004/prepub

## Supplementary Material

Additional file 1 Participatory rural appraisal (PRA) focus group facilitator’s guide outline. Click here for file
