# Supplementary material for: Identification of barriers to the prevention and treatment of heat-related illness in Latino farmworkers using activity-oriented, participatory rural appraisal focus group methods
Source: BMC Public Health. 2013 Oct 24;13:1004. doi: 10.1186/1471-2458-13-1004 (PMC4015616; doi:10.1186/1471-2458-13-1004)
Supplement: Additional file 1 — Participatory rural appraisal (PRA) focus group facilitator’s guide outline. [file 1471-2458-13-1004-S1.doc]

**Additional file: Participatory rural appraisal (PRA) focus group facilitator’s guide outline**

**INTRODUCTION (~15 min)**

- Introduce facilitators, scribe, other project team members
- Explain the purpose of the workshop
- Review consent form, confidentiality, and audio recording
- Let participants know they can ask questions or for assistance at any time

**ACTIVITY 1: Demographics (~10 min)**

- Collect demographic information using electronic audience response system or self-administered on paper

**ACTIVITY 2: Risk factors for and causes of heat-related illness (~15 min)**

- Hand out blank post-it notes
- Ask “What can lead to heat illness?”
- Give 3-5 minutes for participants to write/draw causes on post-it notes and place them on flip-charts
- List main factors on flip chart (environmental factors, activity, personal factors, traditional beliefs, other)
- Go through each cause, ask participants to explain their drawings, and help them place under the correct factor
- Give participants 5 minutes to take a personal risk factors self-assessment, offer help if some participants cannot read

*Post-Activity 2 Education*

- Review risk factors/causes of heat illness after participants finish the self-assessment


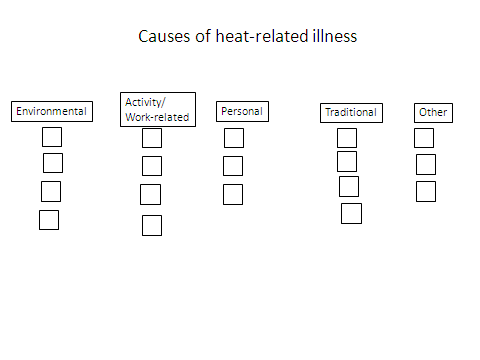


**ACTIVITY 3: Signs and symptoms of heat illnesses (~30 min)**

- Ask a participant to volunteer to lay on the sheet of paper
- Have participants work as a group to make an outline of a body by tracing the shape of one person lying on a large sheet of paper
- Have the participants work as a group to name signs and symptoms of heat illnesses they know from their own or another person’s experience
- Ask participants to write each sign or symptom on a post-it note, attach to the body outline, and draw an arrow to the affected part of the body (make sure all post-it notes are the same color)


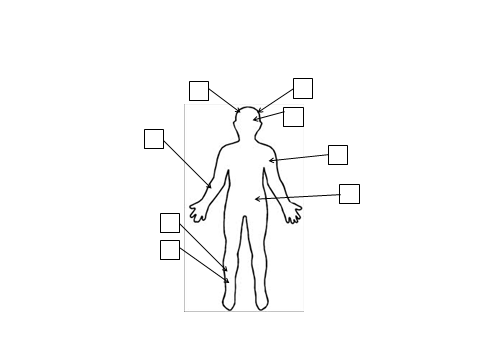


*Post-Activity 3 Education*

- Add the signs and symptoms missing from the participants’ body outline using a different colored post-it note and discuss these signs and symptoms

**ACTIVITY 4: Heat-related illness treatments (~30 min)**

- Label top of flip chart paper with “Heat-related illness treatments”
- Ask “How do you or your family members (your grandmother or mother) treat heat illnesses? These include traditional treatments and what you do in the home. What things do you do, and what things do you not do, and why?”
- Make two columns, one labeled “I do this:” and “I don’t do this:” and one labeled “Why?”
- Record participant answers on flip chart


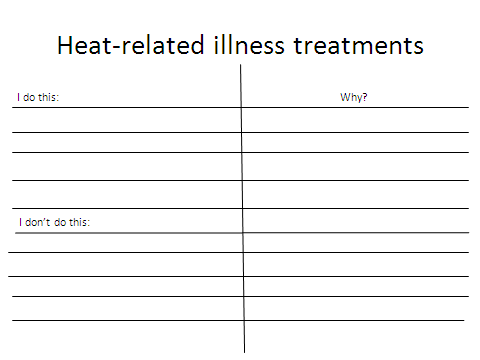


*Post-Activity 4 Education*

- Provide handouts of types of HRI and review recommended treatments for each type of HRI

**ACTIVITY 5: Clothing (~20 min)**

- Look at pictures of workers wearing different types of clothing and describe how the clothing worn may or may not increase the risk of heat illness
- Ask participants to describe the clothing and why specific clothing items may be chosen


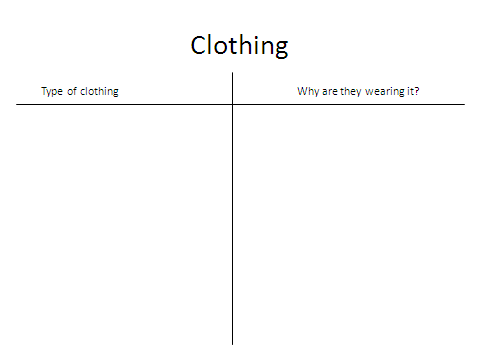


*Post-Activity 5 Education*

- Show picture of person in appropriate clothing and go over recommended clothing
- Have discussion about importance of removing personal protective equipment (PPE) and non-breathable clothing during all breaks

**ACTIVITY 6: Fluids (~20 min)**

- Make flip chart with three columns and label “beverage,” “why do you drink this at work?” and “source”
- Ask participants what beverages they drink at work, why, and where it comes from
- Ask how frequently participants drink water or other acceptable beverages on a hot day and why
- Ask if there are barriers to drinking enough fluids
- Record answers on flip chart


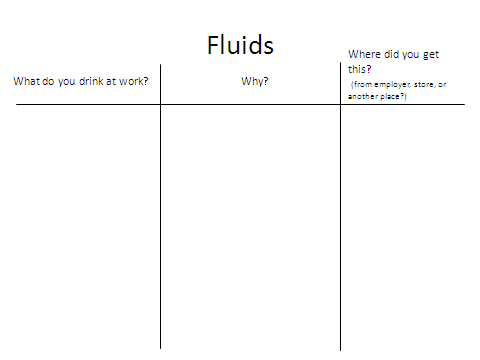


*Post-Activity 6 Education*

- Review what is and is not recommended for hydration
- Review the ½ L water per ½ hour recommendation that should be followed in extreme heat

**ACTIVITY 7: Characteristics of water (~15 min)**

- Ask “what are the five qualities of water most important to you in deciding whether or not you will drink water provided on the job?”
- Make a table, creating a row for each quality but leave the top row blank
- In the same order list the qualities in each column at the top of the table (table with examples below):

- Cross out boxes where the name of the same qualities intersect on the chart and all those that are below the boxes that were crossed out

- Have group discuss which qualities are more important
- Write the name of the quality chosen as most important in the box where the two qualitites intersect
- Continue until all boxes are filled

**ACTIVITY 8: Prevention of heat-related illness – closing educational exercise (~15 min)**

- Ask participants to turn to their neighbor
- Have them tell their neighbor two or three things they learned today about heat illness and prevention and record them
- Collect answers and discuss as a group (possible answers include reporting symptoms to supervisor, proper clothing, keeping hydrated)
- Focus on the worker and what the worker can do (drink water, seek shade, report signs and symptoms of HRI, appropriate emergency response, etc.)
- Emphasize importance of acclimatization
- Discuss other issues that participants bring up, including barriers for HRI prevention
